# The aMAP score improves discrimination of prognostic models in hepatocellular carcinoma after radiofrequency ablation

**DOI:** 10.3389/fonc.2026.1849758

**Published:** 2026-06-17

**Authors:** Luchang Fan, Yiyan Zhang, Jianmin Ding, Yan Zhou, Jun Li, Qiong Wu, Shuqin Cheng, Fengmei Wang, Xiang Jing

**Affiliations:** 1 First Central Hospital of Tianjin Medical University, Tianjin, China; 2 Department of Ultrasound, Tianjin Third Central Hospital, Tianjin, China; 3 Gastroenterology and Hepatology, Tianjin Third Central Hospital, Tianjin, China; 4 Department of Gastroenterology and Hepatology, Tianjin First Central Hospital, Tianjin, China; 5 Tianjin Key Laboratory of Molecular Diagnosis and Treatment of Liver Cancer, Tianjin First Central Hospital, Tianjin, China

**Keywords:** aMAP score, hepatocellular carcinoma, prognostic factor, radiofrequency ablation, recurrence-free survival

## Abstract

**Purpose:**

The aMAP score has proven a reliable model for predicting the risk of hepatocellular carcinoma (HCC) development in patients with various types of hepatitis, but its value in predicting recurrence in HCC patients after radiofrequency ablation (RFA) remains unclear. This study aims to assess the utility of the aMAP score for predicting recurrence in HCC patients after RFA.

**Methods:**

The retrospective analysis included 350 firstly diagnosed HCC patients who underwent RFA. Survival analysis was performed using Kaplan-Meier curves with the log-rank test. Cox regression analysis was conducted to assess the prognostic value of aMAP score. The Fine-Gray method was adopted for sensitivity analysis. Subgroup analyses verified the consistency of the aMAP score’s predictive value. C-index, net reclassification improvement (NRI), and integrated discrimination improvement (IDI) were used to evaluate its incremental predictive value.

**Results:**

Among 350 patients undergoing RFA, a total of 197 (56.3%) patients had a low aMAP score (≤ 63.55), and 153 patients (43.7%) had a high aMAP score (> 63.55). Patients with low aMAP scores exhibited significantly longer recurrence-free survival (RFS) (*p* = 0.016). Multivariate analysis confirmed that the aMAP score is an independent prognostic factor for RFS (*HR* = 1.40, *p* = 0.028). Furthermore, when incorporated into a tumor-related characteristics model (tumor number and size of largest tumor), the aMAP score provided significant incremental value in predicting RFS (IDI 2.6%, *p* = 0.036; NRI 15.9%, *p* = 0.036).

**Conclusions:**

The aMAP score is a potentially predictor for HCC patients undergoing RFA. Incorporation of the aMAP score into the prediction model improved its predictive accuracy.

## 1 Introduction

Hepatocellular carcinoma (HCC) ranks sixth in global incidence and is the third most leading cause of cancer-related deaths worldwide (1–4). Radiofrequency ablation (RFA) serves as a crucial treatment for early-stage HCC, particularly in patients with Barcelona Clinic Liver Cancer Staging (BCLC) stage 0 or A (5–7). However, recurrence-free survival (RFS) and overall survival (OS) remain suboptimal in HCC patients following RFA (8–10). Therefore, identifying reliable prognostic factors for HCC patients following RFA is crucial.

Currently, numerous studies have confirmed that tumor number, size, location, alpha-fetoprotein (AFP) levels, Des-gamma-carboxy prothrombin (DCP) levels, and the albumin−bilirubin (ALBI) grade serve as prognostic predictors for HCC patients who have undergone RFA (11–17). A quantitative model incorporating clinic-radiological factors and intratumoral heterogeneity (ITH) features has been established by Zhang et al. to assess the risk of early recurrence (18). In addition, based on AFP, tumor burden score (TBS) and ALBI grade, Huang et al. constructed the ATA/AT model to facilitate individualized prognosis prediction for HCC patients who undergo curative RFA (15). However, there is no universally accepted standard for predicting recurrence in HCC patients following RFA treatment that is applied in clinical practice.

The aMAP risk score (Age-Male-ALBI-Platelets score) (19) is a recently developed novel assessment model for HCC, designed to evaluate the risk of HCC developing in patients with viral or non-viral hepatitis across diverse ethnicities. This model primarily incorporates five indicators: age, gender, albumin, total bilirubin and platelet counts. Multiple studies have demonstrated that this model exhibits favorable performance in predicting the risk of HCC occurrence (20, 21). In addition, the prognostic value of the aMAP score has been validated in several studies. For instance, the aMAP score has been identified as an independent prognosis factor for intermediate-stage HCC (22); within the Milan criteria, aMAP score can predict the recurrence risk following radical surgical resection in HCC patients (23, 24); it has also been shown to predict early recurrence within one year after microwave ablation for small HCC (25); furthermore, evidence indicates that the aMAP score serves as an independent prognostic factor for late recurrence following RFA (26, 27). Nevertheless, current data on the predictive value of the aMAP score for prognosis after RFA treatment of HCC remains limited. No studies have yet confirmed whether it can improve the predictive value of existing prognostic models, and further research is required to comprehensively evaluate their incremental value in prognostic models.

Based on the foregoing context, this study conducted a retrospective analysis of a real-world cohort of HCC patients. Through Kaplan-Meier curves and Cox proportional hazard regression model, this study confirmed that the aMAP score serves as an independent prognostic factor for recurrence in HCC patients following RFA. Furthermore, using the integrated discrimination improvement (IDI) and net reclassification improvement (NRI) indices, this study validated the incremental predictive value of the aMAP score in HCC recurrence prediction models. This finding not only expands the clinical application scope of the aMAP score but also provides a novel and reliable reference for the precise assessment of post-RFA recurrence risk in HCC patients.

## 2 Methods

### 2.1 Patients

**Figure 1:**
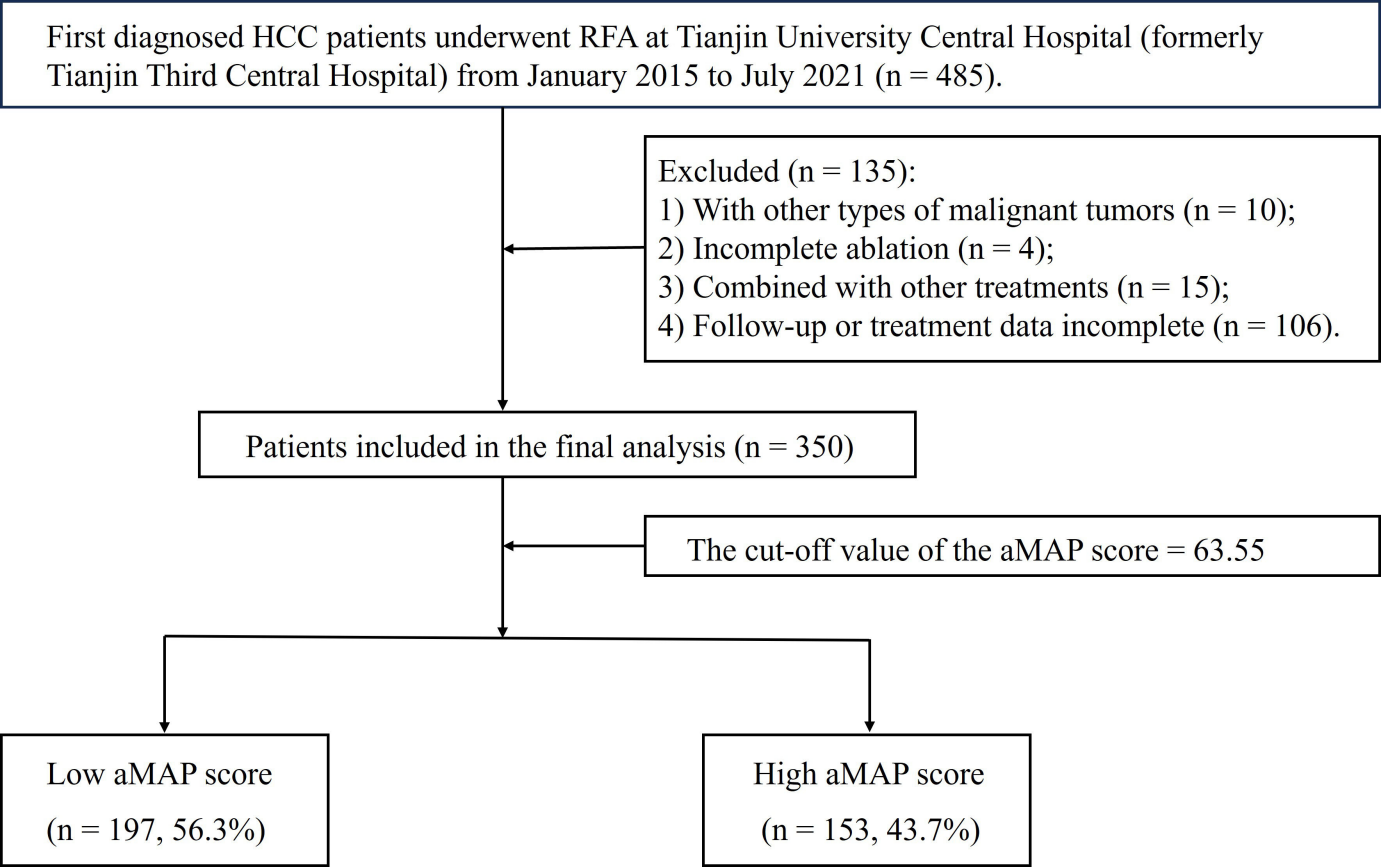
Flow-chart of this study.

This retrospective study included patients treated at Tianjin University Central Hospital (formerly Tianjin Third Central Hospital) between January 2015 and July 2021. All patients were first diagnosed with HCC and underwent RFA as initial treatment. Inclusion criteria included newly diagnosed HCC patients who underwent RFA as initial treatment, with tumor BCLC stage 0 or A and Child-Pugh grade A or B. Exclusion criteria comprised: (1) with other types of malignant tumors; (2) incomplete ablation; (3) combined with other treatments; and (4) follow-up or treatment data incomplete. A total of 135 patients (27.8%) were excluded from the study, including 10 patients (2.1%) with other malignant tumors, 4 patients (0.8%) with incomplete ablation, 15 patients (3.1%) receiving combined antitumor therapy, and 106 patients (21.9%) with incomplete follow-up or treatment data. Ultimately, 350 patients were enrolled in this study. The flow chart of the study is shown in **Figure 1**.

This observational study was approved by the Ethics Committee of Tianjin University Central Hospital (formerly Tianjin Third Central Hospital) and conducted in accordance with the Declaration of Helsinki. All patients provided verbally informed consent for the retrospective use of their anonymized data in health-related research, as approved by the Ethics Committee.

### 2.2 Data collection

Patient data on demographic characteristics, medical history, personal history, treatment details, and follow-up were collected by reviewing the electronic medical records system. Venous blood samples were drawn after overnight fasting prior to RFA treatment. The levels of red blood cell (RBC), white blood cell (WBC), hemoglobin (HGB), platelet (PLT), alanine aminotransferase (ALT), aspartate aminotransferase (AST), albumin (ALB), total bilirubin (TBIL), alkaline phosphatase (ALP), γ-glutamyl transpeptidase (γ-GT), international normalized ratio (INR) and alpha-fetoprotein (AFP) were measured.

### 2.3 aMAP score

The aMAP score for each HCC patient was calculated using the following formula: [(age [years] × 0.06 + sex × 0.89 (male: 1, female: 0) + (log_10_ bilirubin [μmol/L] × 0.66 - albumin [g/L] × 0.085) × 0.48 − 0.01 × platelet count [10^3^/mm^3^] + 7.4]/14.77 × 100 (19).

### 2.4 Follow-up

Following RFA, the patient was advised to attend regular follow-up visits as guided by their physicians. Specifically, patients underwent post-treatment evaluation 1–2 months after radiofrequency ablation (RFA), including imaging examinations and serum tumor markers, to assess the completeness of tumor ablation. Regular follow-up was subsequently performed every 3 months during the first two years and every 3–6 months thereafter. All participants were followed up until tumor recurrence, loss to follow-up, or the end of the study. The final follow-up date in this study was June 1, 2024.

RFS was defined as the period from the date of ablation to the date of tumor recurrence or the last follow-up date. OS was defined as the period from the date of ablation to death from any cause. Complete ablation was defined as the absence of residual tumor tissue confirmed by contrast-enhanced ultrasound, contrast-enhanced computed tomography, or other appropriate imaging performed one month after RFA.

### 2.5 Statistical analysis

First, the optimal cutoff value for the aMAP score was determined using the surv_cutpoint function from the survminer R package. Subsequently, the robustness of this cutoff was evaluated through 1,000 bootstrap resampling iterations, and the median cutoff derived from the bootstrap distribution was adopted as the final threshold to reduce the risk of overfitting. Continuous variables were presented as mean ± standard deviation (SD) or median (interquartile range, IQR) and analyzed using Student’s t-test or Mann-Whitney U test. Categorical variables are presented as number (percentage) and were compared using the chi-square test. Kaplan-Meier curves stratified by aMAP score cutoff, with log-rank test for group differences. The cox proportional hazard model was employed to evaluate the prognostic value of aMAP score for OS and RFS. In the sensitivity analysis, the Fine-Gray method was used to estimate the cumulative incidence function (CIF), and Gray’s test was applied to compare differences between groups. A multivariable Fine–Gray competing risks regression model was further constructed to assess the independent effect of each covariate on the risk of recurrence. Variance inflation factor (VIF) was used to assess multicollinearity. Subgroup analyses were performed to evaluate the consistency of the aMAP score’s predictive value for RFS. The incremental predictive value of incorporating the aMAP score was assessed using C-statistics, integrated IDI and NRI. All statistical analysis was performed using R software (version 4.4.3; R Foundation for Statistical Computing, Vienna, Austria). Differences were considered significant at *p* < 0.05.

## 3 Results

### 3.1 Characteristics of patient cohort

A total of 350 patients were enrolled. The median aMAP score in the entire cohort was 62.68 (IQR 58.41 - 67.02). The optimal cutoff value for the aMAP score was 63.55 (**Supplementary Figure 1**). Bootstrap resampling analysis demonstrated good robustness of this cutoff, with a median of 63.55 (95% CI: 55.47–68.57). Based on this threshold, 197 patients (56.3%) were classified into the low-risk group and 153 patients (43.7%) into the high-risk group. **Table 1** shows the general characteristics of the study population.

**Table 1:** Clinical characteristics of the entire cohort.

Variables	Total	Low aMAP score (197, 56.3%)	High aMAP score (153, 43.7%)	*p*
Gender (n, %)				< 0.001
Male	244 (69.7)	119 (60.4)	125 (81.7)	
Female	106 (30.3)	78 (39.6)	28 (18.3)	
Age (years) (M, IQR)	60.00 [54.25 - 65.00]	57.00 [51.00 - 62.00]	63.00 [59.00 - 69.00]	**< 0.001**
Hypertension (n, %)				0.296
No	269 (76.9)	156 (79.2)	113 (73.9)	
Yes	81 (23.1)	41 (20.8)	40 (26.1)	
Diabetes (n, %)				0.232
No	279 (79.7)	162 (82.2)	117 (76.5)	
Yes	71 (20.3)	35 (17.8)	36 (23.5)	
HBV infection (n, %)				
No	67 (19.1)	30 (15.2)	37 (24.2)	**0.048**
Yes	283 (80.9)	167 (84.8)	116 (75.8)	
HCV infection (n, %)				1.000
No	314 (89.7)	177 (89.8)	137 (89.5)	
Yes	36 (10.3)	20 (10.2)	16 (10.5)	
Smoking history				
No	178 (50.9)	109 (55.3)	69 (45.1)	0.073
Yes	172 (49.1)	88 (44.7)	84 (54.9)	
Alcohol history				
No	218 (62.3)	136 (69.0)	82 (53.6)	**0.004**
Yes	132 (37.7)	61 (31.0)	71 (46.4)	
Child-Pugh class (n, %)				0.001
A	309 (88.3)	184 (93.4)	125 (81.7)	
B	41 (11.7)	13 (6.6)	28 (18.3)	
BCLC stage (n, %)				0.662
0	118 (33.7)	64 (32.5)	54 (35.3)	
A	232 (66.3)	133 (67.5)	99 (64.7)	
Tumor number (n, %)				0.471
Single	281 (80.3)	155 (78.7)	126 (82.4)	
Multiple	69 (19.7)	42 (21.3)	27 (17.6)	
Tumor size of largest nodule (cm, n, %)				0.533
< 2.5	212 (60.6)	116 (58.9)	96 (62.7)	
≥ 2.5	138 (39.4)	81 (41.1)	57 (37.3)	
RBC (10^9^/L) (M, IQR)	4.31 [3.93 - 4.69]	4.52 [4.14 - 4.90]	4.11 [3.72 - 4.44]	**< 0.001**
WBC (10^9^/L) (M, IQR)	4.24 [3.10 - 5.39]	4.58 [3.75 - 5.75]	3.41 [2.63 - 4.73]	**< 0.001**
HGB (g/L) (M, IQR)	135.00 [123.25 - 148.00]	140.00 [130.00 - 152.00]	130.00 [117.00 - 141.00]	**< 0.001**
PLT (10^9^/L) (M, IQR)	97.50 [69.00 - 144.00]	128.00 [91.00 - 176.00]	72.00 [56.00 - 94.00]	**< 0.001**
ALT (U/L) (M, IQR)	24.00 [17.00 - 33.00]	24.00 [17.00 - 33.00]	22.00 [17.00 - 32.00]	0.383
AST (U/L) (M, IQR)	26.50 [19.00 - 37.00]	24.00 [19.00 - 34.00]	31.00 [22.00 - 42.00]	**< 0.001**
ALB (U/L) (M, IQR)	41.60 [37.70 - 45.27]	44.30 [40.10 -46.50]	38.60 [34.20 -42.10]	**< 0.001**
TBIL (μmol/L) (M, IQR)	16.30 [12.00 - 23.80]	13.90 [10.60 - 18.70]	19.80 [14.50 -27.90]	**< 0.001**
ALP (U/L) (M, IQR)	82.00 [65.00 - 103.00]	78.00 [64.00 - 99.00]	85.00 [69.00 - 107.00]	**0.046**
γ-GT (U/L) (M, IQR)	38.00 [27.00 - 66.00]	36.00 [24.00 - 57.00]	45.00 [30.00 - 85.00]	**0.001**
INR (M, IQR)	1.09 [1.00 - 1.21]	1.04 [0.99 - 1.11]	1.18 [1.06 - 1.33]	**< 0.001**
AFP (ng/mL) (M, IQR)	9.04 [3.36 - 53.92]	7.75 [2.91, 73.20]	11.20 [4.09, 41.54]	0.493

M, Median; IQR, Interquartile Range; HBV, hepatitis B virus; HCV, hepatitis C virus; BCLC, Barcelona Clinic Liver Cancer; RBC, Red blood cell; WBC, White blood cell; HGB, Hemoglobin; PLT, Platelet; ALT, Alanine aminotransferase; AST, Aspartate aminotransferase; ALB, Albumin; TBIL, Total bilirubin; ALP, Alkaline phosphatase; γ-GT, γ-glutamyl transpeptidase; INR, International Normalized Ratio; AFP, Alpha-fetoprotein.

p values in bold are < 0.05.

### 3.2 Association of aMAP score with clinical characteristics

Patients with high aMAP scores were older (median age: 63 vs 57 years), more frequently male (81.7% vs 60.4%), had alcohol history (46.4% vs 31.0%), had higher levels of AST (median AST: 31 vs 24 U/L), bilirubin (median TBIL: 19.80 vs 13.90 μmol/L), ALP (median ALP: 85.00 vs 78.00 U/L), γ-GT (median γ-GT: 45.00 vs 36.00 U/L) and INR (median INR: 1.18 vs 1.04) compared to the low aMAP group (all *p* < 0.05). Patients with low aMAP scores more frequently had a history of hepatitis B virus (HBV) infection (84.8% vs 75.8%), better Child-Pugh class (93.4% vs 81.7%) and higher levels of RBC, WBC, HGB, PLT, and ALB levels (all *p* < 0.05). In contrast, no significant association was observed between aMAP score and hypertension, diabetes, a history of hepatitis C virus (HCV) infection, smoking history, BCLC stage, AFP level, tumor numbers and size of largest tumor (all *p* > 0.05) (**Table 1**).

### 3.3 Survival analysis

**Figure 2:**
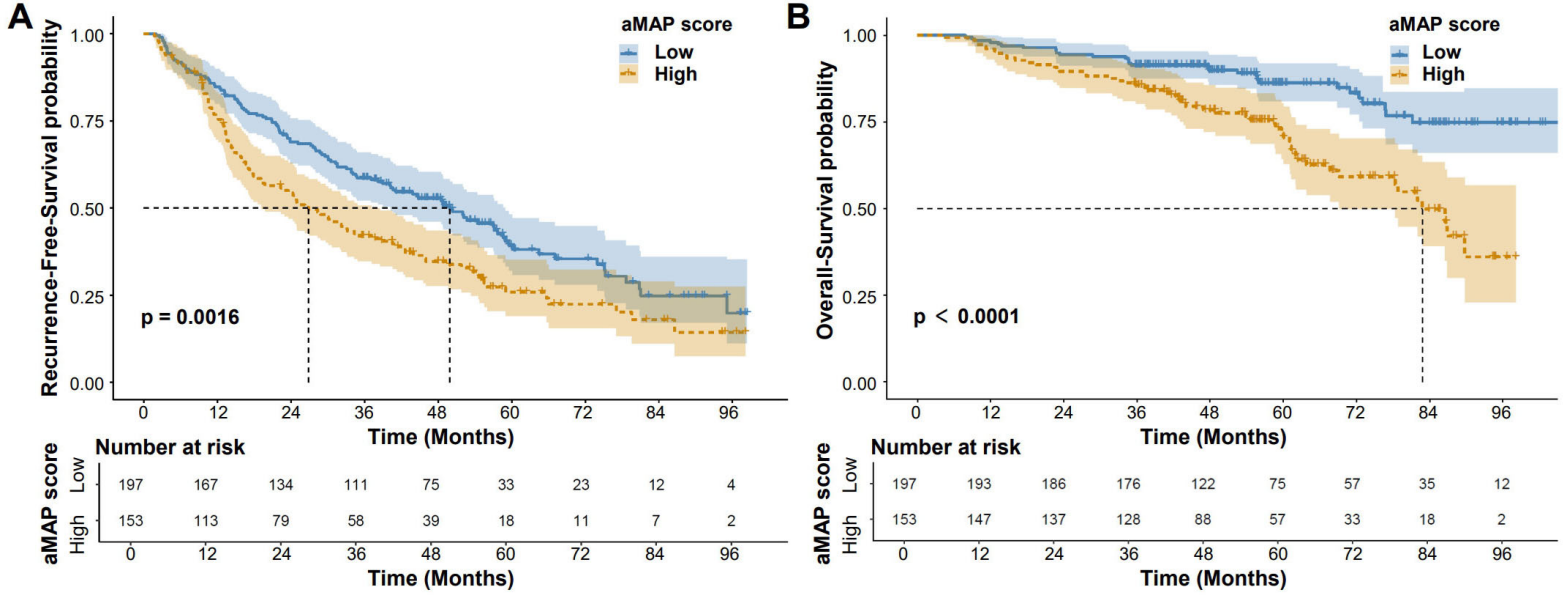
Kaplan-Meier curves demonstrating differences in RFS **(A)** and OS **(B)** among patients with low versus high aMAP score. Time on the x-axis represents months of observation.

The median follow-up times was 35.20 months (IQR 13.85 - 53.24). Kaplan-Meier analysis shows that patients with a low aMAP score had a median RFS of 39.53 months (IQR 20.83 - 55.67), compared with 24.87 months (IQR 11.43 - 48.00) in those with a high aMAP score. Patients with low aMAP scores had significantly longer RFS than those with high aMAP scores (1-year RFS: 84.9% vs 73.9%; 3-year RFS: 56.3% vs 37.9%; 5-year RFS: 16.8% vs 11.8%; *p* = 0.016, **Figure 2A**). Additionally, the aMAP score was a significant predictor of OS following RFA (1-year OS: 98.0% vs 96.1%; 3-year OS: 89.3% vs 83.7%; 5-year OS: 38.1% vs 37.3%; *p* < 0.001, **Figure 2B**). Cumulative incidence curves based on the competing risk model demonstrated that patients in the high-risk group had a significantly higher cumulative incidence of recurrence compared with those in the low-risk group (Gray’s test, *p* = 0.008). In contrast, no statistically significant difference was observed in the cumulative incidence of death between the two groups (*p* = 0.053) (**Supplementary Figure 2**).

### 3.4 The aMAP score is an independent prognostic factor of RFS

In the univariate analysis, RFS was significantly associated with the aMAP score (hazard ratio [HR] = 1.52, 95% confidence interval [CI] 1.17-1.98, *p* = 0.002). All variables had VIF values < 5, indicating no multicollinearity among them (**Supplementary Figure 3**). After adjusting for all potential confounding factors, a high aMAP score was independently associated with an increased recurrence risk (HR = 1.40, 95% CI 1.04-1.89, *p* = 0.028) (**Table 2**). In the Fine–Gray competing risks model, with death prior to recurrence treated as a competing event, the aMAP score remained an independent predictor of recurrence (HR = 1.47, 95% CI: 1.12–1.92, *p* = 0.005) (**Supplementary Table 1**). However, multivariate analysis revealed that the aMAP score was not an independent risk factor for OS (**Supplementary Table 2**).

**Table 2:** Cox regression analysis of factors associated with recurrence-free survival.

Variables	Univariate analysis		Multivariate analysis	
	HR (95% CI)	*p*	HR (95% CI)	*p*
Gender	0.85 (0.64 - 1.13)	0.266		
Age (years)	1.00 (0.99 - 1.02)	0.621		
Hypertension	1.04 (0.76 - 1.43)	0.790		
Diabetes	0.98 (0.71 - 1.35)	0.908		
HBV infection	1.18 (0.84 - 1.65)	0.350		
HCV infection	0.63 (0.4 - 1.00)	0.051		
Smoking history	1.30 (1.00 - 1.68)	0.053		
Alcohol history	1.18 (0.90 - 1.54)	0.240		
Child-Pugh class	1.10 (0.74-1.65)	0.629		
BCLC stage	1.44 (1.08-1.91)	**0.013**	1.14 (0.80-1.63)	0.464
Tumor number	1.75 (1.28-2.38)	**0.000**	1.78 (1.28-2.49)	**0.001**
Tumor size of largest nodule (cm)	1.42 (1.08-1.85)	**0.010**	1.44 (1.05-1.97)	**0.024**
RBC (10^9^/L)	0.95 (0.76-1.18)	0.632		
WBC (10^9^/L)	0.98 (0.90-1.06)	0.541		
PLT (10^9^/L)	1.00 (1.00-1.01)	0.123		
HGB (g/L)	1.00 (1.00-1.01)	0.269		
AST (U/L)	1.01 (1.00-1.01)	**0.001**	1.00 (1.00-1.01)	0.234
ALT (U/L)	1.01 (1.00-1.01)	**0.011**	1.00 (0.99-1.01)	0.550
ALP (U/L)	1.00 (1.00-1.01)	0.107		
ALB (U/L)	0.96 (0.94-0.99)	**0.005**	1.00 (0.97-1.03)	0.791
TBIL (μmol/L)	1.01 (1.00-1.02)	0.132		
γ-GT (U/L)	1.00 (1.00-1.01)	**0.009**	1.00 (1.00-1.00)	0.579
INR	2.58 (1.25-5.32)	**0.010**	1.76 (0.66-4.69)	0.256
aMAP score	1.52 (1.17-1.98)	**0.002**	1.40 (1.04-1.89)	**0.028**
AFP (ng/mL)	1.00 (1.00-1.01)	0.266		

HR, Hazard Ratio; CI, Confidence Interval; HBV, hepatitis B virus; HCV, hepatitis C virus; BCLC, Barcelona Clinic Liver Cancer; RBC, Red blood cell; WBC, White blood cell; HGB, Hemoglobin; PLT, Platelet; ALT, Alanine aminotransferase; AST, Aspartate aminotransferase; ALB, Albumin; TBIL, Total bilirubin; ALP, Alkaline phosphatase; γ-GT, γ-glutamyl transpeptidase; INR, International Normalized Ratio; AFP, Alpha-fetoprotein.

*p* values in bold are < 0.05.

### 3.5 Subgroup analysis

**Figure 3:**
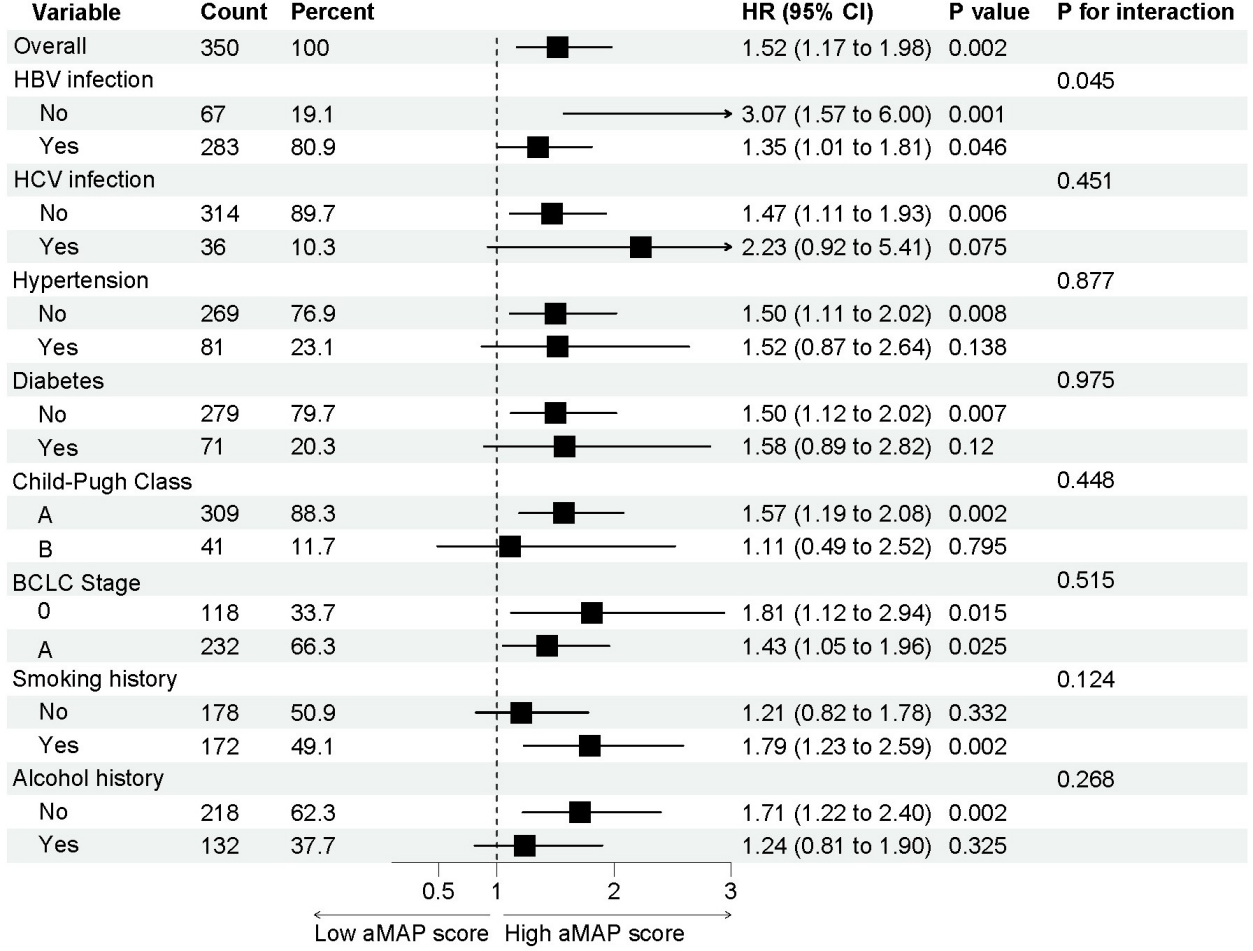
Subgroup analyses showing the effect of aMAP score on RFS across different subgroups defined by HBV/HCV infection, hypertension, diabetes, Child-Pugh class, BCLC stage, smoking history and alcohol history.

The subgroups analyses revealed heterogeneity in the association across subgroups on HCV infection, hypertension, diabetes, Child-Pugh class, smoking and alcohol history. A significant interaction was observed between HBV infection and the aMAP-RFS association (*p* < 0.05). Further analysis demonstrated that a consistent association between aMAP score and RFS remained robust in patients without HCV infection, hypertension, diabetes or alcohol history. Additionally, in patients with Child-Pugh A and patients with smoking history, RFS was significantly associated with the aMAP score. Of note, the aMAP score was strongly associated with RFS regardless of whether patients were at BCLC stage 0 or A (**Figure 3**).

### 3.6 The incremental predictive value of aMAP score

Three distinct models were constructed to evaluate the potential role of the aMAP score in predicting recurrence. The basic model included tumor-related characteristics (including tumor number and size of largest nodule). The second model incorporated all the aforementioned variables plus aMAP score treated as a continuous variable. The third model replaced the continuous aMAP score with its categorical counterpart. This sequential model construction facilitates comparisons of predictive accuracy and interpretability by assessing whether the addition of the aMAP score (either as a continuous or categorical variable) significantly enhances recurrence prediction beyond the traditional prognostic factors of tumor number and largest tumor nodule size. The results are summarized in **Table 3**. Based on the C-index, NRI, and IDI, the addition of the categorical aMAP score to the baseline model significantly improved the predictive performance for recurrence (C-index: 0.582 vs. 0.616, ΔC = 0.034, *P* = 0.032; IDI: 2.6%(0.1%-7.3%); *p* = 0.036; NRI: 15.9%(1.0%-32.6%); *p* = 0.036). In contrast, the incorporation of the continuous aMAP score into the baseline model did not yield a statistically significant improvement in predictive accuracy (C-index: 0.582 vs. 0.603, ΔC = 0.021, *P* = 0.052; IDI: 1.9%(-0.2%-6.4%); *p* = 0.096; NRI: 7.6%(-14.7%-23.1%); *p* = 0.483). This study also compared the ALBI-combined baseline model with Model 3, and the results demonstrated that adding the categorical aMAP score to the baseline model was superior to adding ALBI (**Supplementary Table 3**). This indicates that incorporating the categorical aMAP score can enhance the performance of the model.

**Table 3:** Comparison of discrimination for a new model including tumor characteristics and aMAP score in respect to tumor characteristics alone.

Variable	Basic model		Basic model + continuous aMAP score		Basic model + categorical aMAP score	
	HR (95% CI)	*p*	HR (95% CI)	*p*	HR (95% CI)	*p*
tumor number	1.81 (1.33 - 2.47)	**< 0.001**	1.76 (1.29 - 2.40)	**< 0.001**	1.84 (1.35 - 2.50)	**< 0.001**
tumor size of largest nodule	1.46 (1.12 - 1.91)	**0.005**	1.50 (1.15 - 1.96)	**0.003**	1.48 (1.14 - 1.93)	**0.004**
aMAP score	–	–	1.03 (1.01 - 1.05)	**0.015**	1.57 (1.21 - 2.04)	**0.001**
C-index	0.582 (0.543-0.620)	Ref.	0.603 (0.564-0.643)	0.052	0.616 (0.579-0.654)	**0.032**
IDI	Ref.		1.9% (-0.2%-6.4%)	0.096	2.6% (0.1%-7.3%)	**0.036**
NRI	Ref.		7.6% (-14.7%-23.1%)	0.483	15.9% (1.0%-32.6%)	**0.036**

NRI net reclassification improvement; IDI integrated discrimination improvement.

P values in bold are < 0.05.

## 4 Discussion

This analysis reveals three main findings: 1) The aMAP score is an independent prognostic factor for patients with HCC following RFA. 2) Patients with a concomitantly high aMAP score demonstrated a significantly increased risk of recurrence compared to those with a low score. 3) The aMAP score reliably predicts prognosis and improves the predictive accuracy beyond that of traditional factors, such as tumor number and size.

The aMAP score, a model first developed by Fan et al., is a validated tool for predicting the development of HCC across diverse etiologies and ethnicities (19). The aMAP score consists of the albumin/bilirubin ratio, platelet count, age and sex. The albumin-bilirubin score represents a simple and objective index that reflects the underlying liver function (28–30). In addition, platelet count is closely associated with fibrosis stage. To be specific, platelets can influence liver fibrosis by interactions with liver sinusoidal endothelial and hepatic stellate cells (31–33). Supporting its broad clinical applicability, a meta-analysis has validated - the aMAP score as a reliable, accurate, and user-friendly tool for HCC risk prediction across all patients’ populations (20). Its clinical utility is further corroborated by real-world evidence (21). The aMAP score has demonstrated clinical promise for forecasting HCC occurrence in specific patients, including HBV infection patients receiving entecavir (ETV) or tenofovir disoproxil fumarate (TDF) antiviral regimens (34, 35) and HCV infection patients after sustained virologic response (SVR) (36). Notably, combining aMAP score with liver stiffness (LS) assessment enhances HCC risk stratification in CHB patients receiving antiviral therapy (37).

Beyond serving as a tool for HCC surveillance, the aMAP score has also emerged as a valuable, non-invasive biomarker for assessing liver fibrosis evaluation in patients with CHB. A combined model incorporating the aMAP score and liver stiffness measurement (LSM) has demonstrated accurate fibrosis staging in treated individuals (38). Elevated aMAP scores independently predict adverse clinical outcomes in hepatitis B virus-related acute-on-chronic liver failure (HBV-ACLF) patients who survive beyond six months (39). Recently, much research has shown its utility in forecasting postoperative outcomes following hepatic resection of HCC both within and outside the Milan criteria (23, 24). In another single-center study from China, Chen et al. further identified the aMAP score as an independent risk factor for intermediate-stage HCC (22). Moreover, the aMAP score reliably predicts early recurrence within one year after microwave ablation for small HCC (25). It also independently predicts late recurrence (LR) of HCC following RFA (26) and serves as an objective prognostic indicator of LR in patients with HBV-related HCC after RFA (27). As key components of the aMAP score, the ALBI grade and platelet count have been proven useful for predicting HCC recurrence (40–42). Due to variations in study designs and populations, the optimal cut-off value of aMAP scores for predicting HCC prognosis shows heterogeneity across studies. In the present analysis, the optimal cut-off for discriminating prognosis in HCC patients undergoing RFA was determined to be 63.55.

Building upon previous literature, the present study confirmed that the aMAP score was independently associated with post-RFA recurrence in HCC patients. Consistent with previous studies (22, 24, 26), patients with higher aMAP scores exhibited poorer OS and RFS compared to those with lower aMAP scores (**Figures 2A, B**). Additionally, individuals with HBV infection tend to have lower aMAP scores. Previous research has shown that persistent HBV triggers chronic liver inflammation, induces hepatocyte injury, activating hepatic stellate cells, and promotes the synthesis and deposition of substantial extracellular matrix, ultimately leading to liver fibrosis (43, 44). This may be because all HBV-related HCC patients included in this study had undergone standardized antiviral therapy, which effectively suppressed viral replication, reduced hepatocellular inflammation and necrosis, and significantly improved hepatic synthesis and metabolic functions—resulting in relatively low aMAP scores. Subgroup analysis revealed a significant interaction between HBV infection status and aMAP score with respect to HCC recurrence risk. Specifically, in patients without HBV infection, the aMAP score exhibited substantially stronger predictive performance for recurrence risk (HR = 3.07), whereas this association was markedly attenuated in those with HBV infection (HR = 1.35). Previous studies have confirmed the efficacy of the aMAP score in predicting HCC recurrence risk in HBV-infected populations (27). This aligns with our findings that the aMAP score remains significantly associated with recurrence in patients with HBV infection (*p* = 0.046), thereby further validating its utility for assessing recurrence risk in HBV-related HCC. Notably, this study further revealed that HBV infection status significantly modulates the predictive efficacy of the aMAP score (interaction *p* = 0.045): specifically, the strength of the association between the aMAP score and recurrence risk was substantially higher in non-HBV-infected patients compared with those with HBV infection (HR = 3.07 vs 1.35). This observation underscores the “population specificity” of the aMAP score, implying that it may exhibit superior predictive performance in non-HBV-infected cohorts. Further validation in larger sample sizes is warranted.

In this study, we further assessed the incremental predictive value of adding the aMAP score to existing models using the IDI and NRI. The inclusion of the aMAP score yielded a significant improvement, with an NRI of 0.159 (*p* < 0.05) and an IDI of 0.026 (*p* < 0.05) (**Table 3**). Notably, the aMAP score was not associated with tumor intrinsic factors including tumor number and size of the largest tumor (**Table 1**), indicating it reflects patient-specific characteristics independent of tumor features. Incorporating the aMAP score with conventional tumor-related factors (tumor number and size of the largest tumor) significantly enhances the predictive accuracy for post-RFA tumor progression. Given its non-invasive nature, ease of determination, repeatability, and low cost, the aMAP score serves as a valuable tool to optimize recurrence risk stratification in clinical practice.

Regarding OS in HCC, previous studies have established the aMAP score as a predictor of OS in patients receiving curative treatment such as surgical resection and transcatheter arterial chemoembolization (TACE) (22, 23). However, its predictive value for OS following RFA remains scarcely explored. In our study, patients with a low aMAP score had a significantly higher OS than those with a high score (*p* < 0.001). Nevertheless, multivariate analysis indicated that the aMAP score was not an independent risk factor for OS. This lack of independent association may be attributed to several factors: OS is a complex endpoint influenced by post-procedural treatments, comorbidities, and non-tumor-related mortality. Furthermore, the retrospective nature of the study may introduce potential information bias.

This study has several limitations. First, its retrospective design may introduce selection bias and other unintended confounding factors. Second, the relatively small sample size from a single center may limit the generalizability of our findings; additionally, the optimal cutoff value of the aMAP score was determined and subsequent analyses were conducted within the same dataset, which could introduce circular validation bias and inflate the Type I error rate. Although we adopted 1000-times Bootstrap resampling to improve robustness and reduce overfitting risk, such Bootstrap-based internal validation is still limited, and external validation in independent cohorts is warranted. Third, deviations from the prescribed postoperative surveillance protocol could have affected the timing and accuracy of recurrence detection. Lastly, as this study exclusively analyzed patients undergoing RFA for HCC, the results may not be generalizable to those receiving other treatments.

## 5 Conclusions

In conclusion, our study validates the aMAP score as a potentially predictor for HCC patients undergoing RFA. Its incorporation enhances the predictive accuracy of conventional clinical factors such as tumor number and size. Given its non-invasive nature, ease of determination, repeatability, and low cost, the aMAP score represents a promising candidate tool for clinical recurrence risk assessment.

## Data Availability

The original contributions presented in the study are included in the article/**Supplementary Material**. Further inquiries can be directed to the corresponding author.
