# Supplementary material for: The aMAP score improves discrimination of prognostic models in hepatocellular carcinoma after radiofrequency ablation
Source: Front Oncol. 2026 Jun 17;16:1849758. doi: 10.3389/fonc.2026.1849758 (PMC13318713; doi:10.3389/fonc.2026.1849758)
Supplement: Supplementary file 1 [file DataSheet1.docx]

Supplementary Material


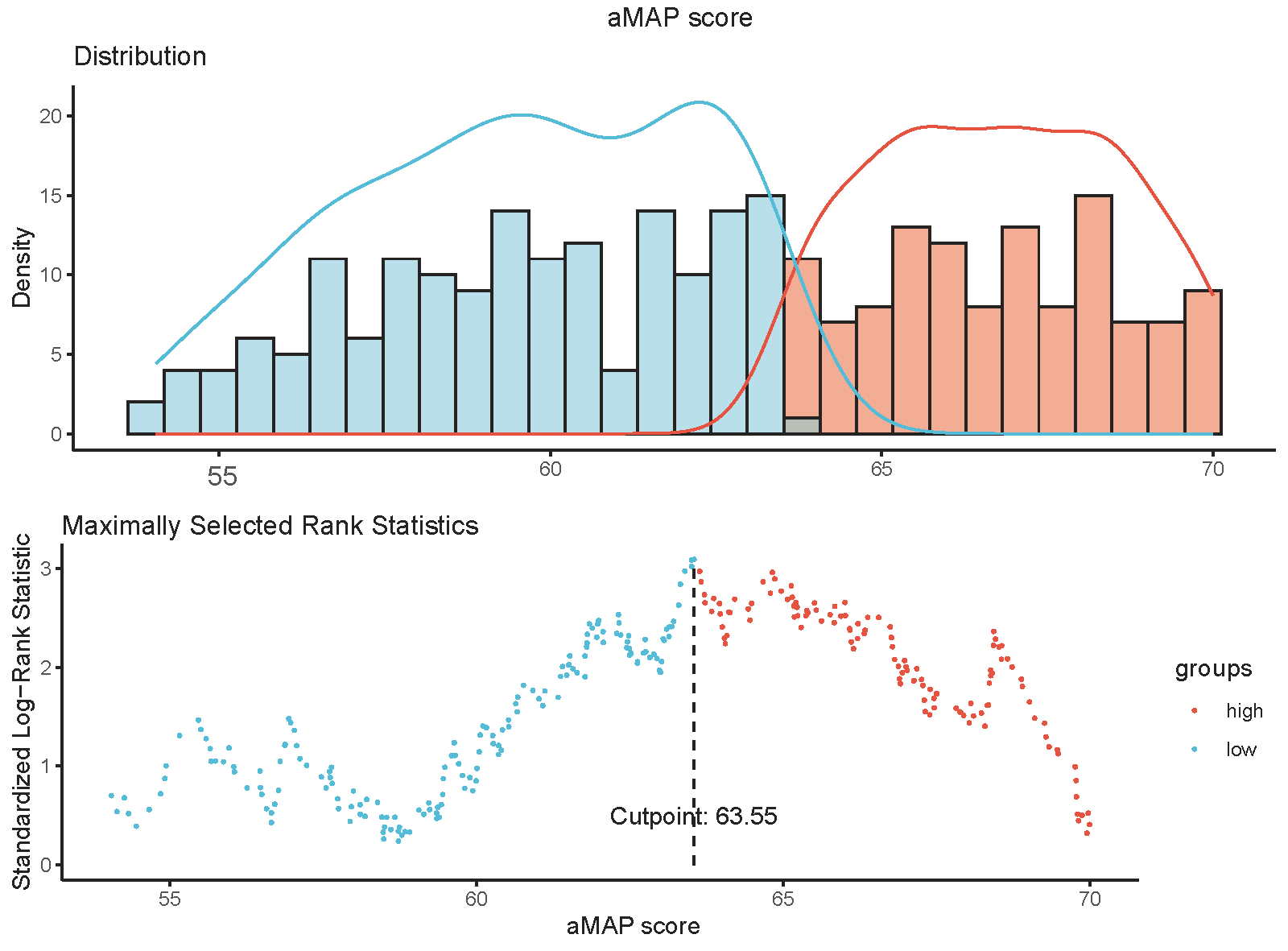


**Supplementary Figure 1.** The data distribution after grouping based on the optimal cutpoint.


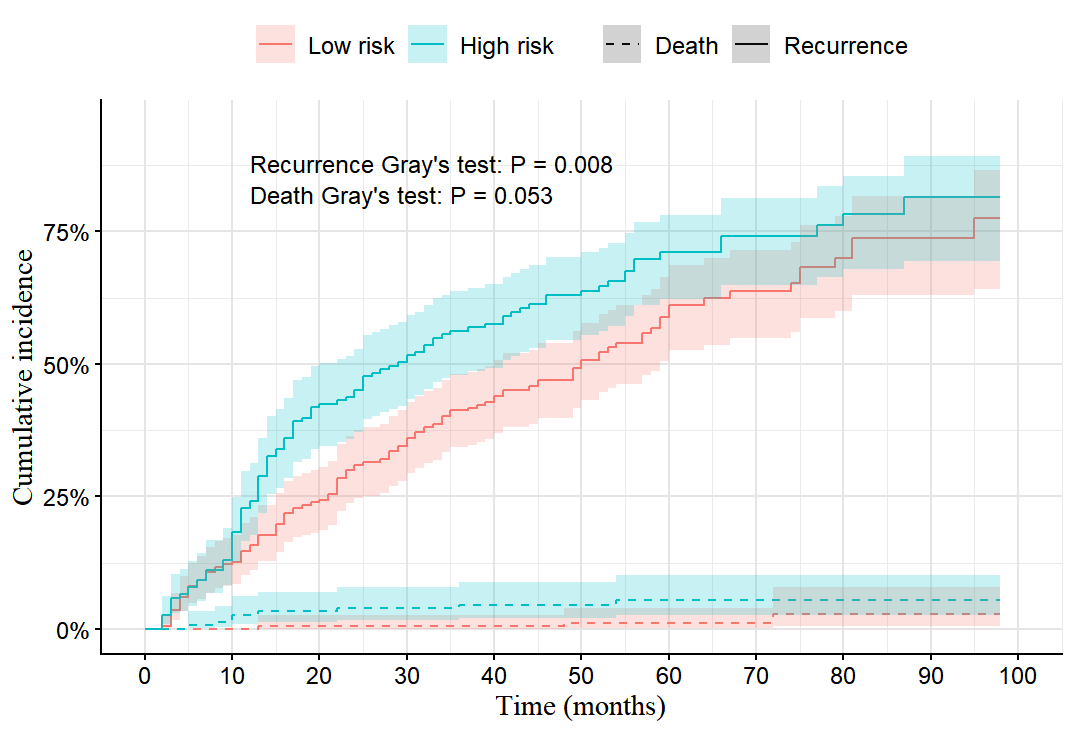


**Supplementary Figure 2.** Cumulative incidence curves for recurrence and death according to aMAP risk groups. The cumulative incidence of recurrence (solid lines) and death (dashed lines) was estimated using the Fine–Gray method. Differences between groups were compared using Gray’s test. The high-risk group showed a significantly higher incidence of recurrence compared with the low-risk group (*p* = 0.008), whereas no significant difference was observed for death (*p* = 0.053).


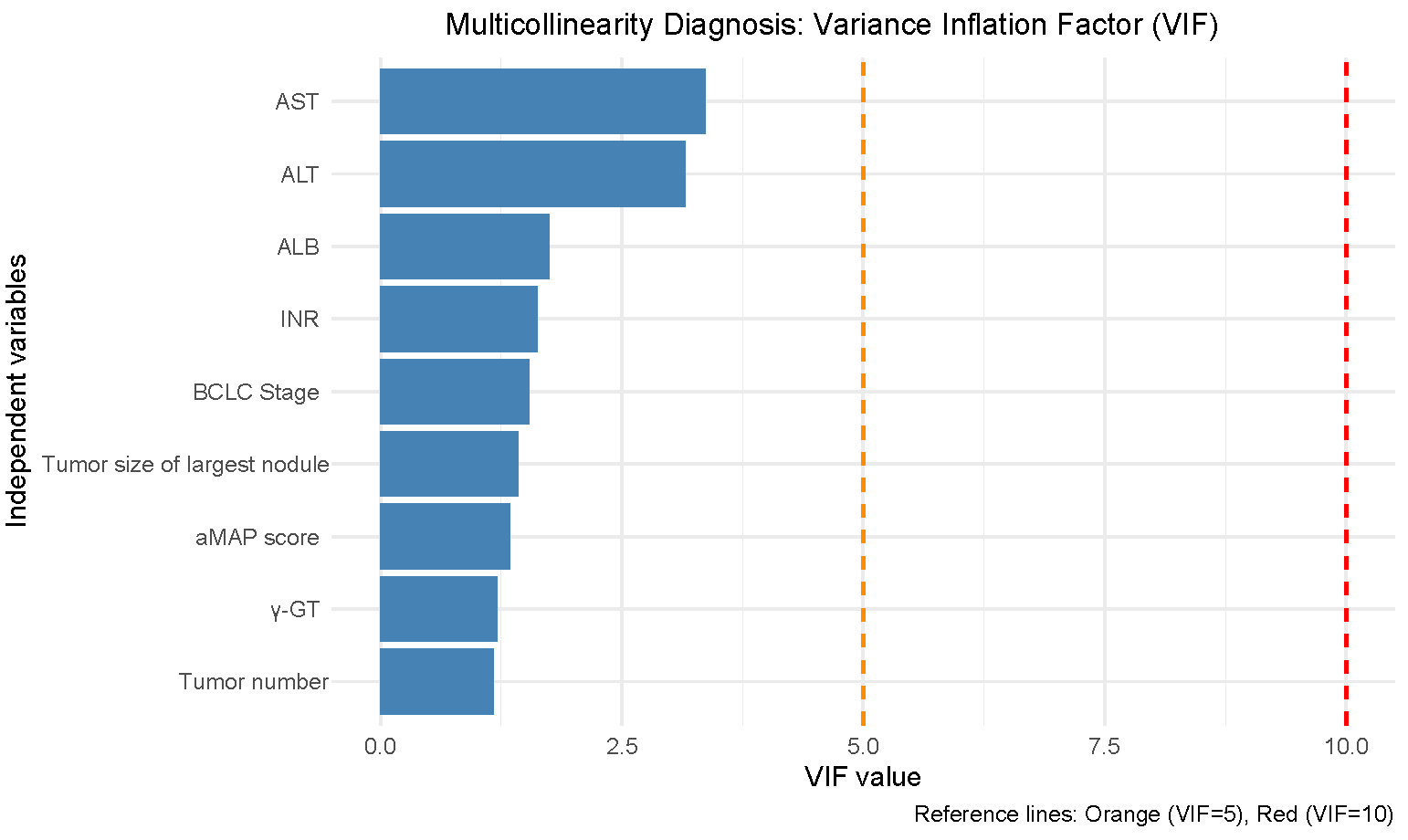


**Supplementary Figure 3.** Variance Inflation Factor (VIF) for multicollinearity diagnosis and visualization.

**Supplementary Table 1.** Multivariable Fine-Gray competing risks regression analysis for recurrence-free survival.

| **Variables** | **HR (95% CI)** | ***p*** |
| --- | --- | --- |
| aMAP score | 1.47 (1.12–1.92) | 0.005 |
| Tumor number | 1.82 (1.31–2.54) | <0.001 |
| Tumor size of largest nodule | 1.40 (1.06–1.83) | 0.016 |

**Supplementary Table 2.** Cox regression analysis of factors associated with overall-survival.

| **Variables** | **Univariate analysis** | |  | **Multivariate analysis** | |
| --- | --- | --- | --- | --- | --- |
|  | HR (95% CI) | *p* |  | HR (95% CI) | *p* |
| Gender | 0.59 (0.34 - 1.00) | **0.049** |  | 0.79 (0.41 - 1.51) | 0.473 |
| Age (years) | 1.00 (0.97 - 1.02) | 0.752 |  |  |  |
| Hypertension | 0.87 (0.51 - 1.49) | 0.613 |  |  |  |
| Diabetes | 0.97 (0.57 - 1.65) | 0.904 |  |  |  |
| HBV infection | 1.51 (0.82 - 2.78) | 0.190 |  |  |  |
| HCV infection | 0.26 (0.08 - 0.82) | **0.022** |  | 0.24 (0.07 - 0.77) | **0.017** |
| Smoking history | 1.66 (1.07 - 2.57) | **0.024** |  | 1.25 (0.74 - 2.14) | 0.406 |
| Alcohol history | 1.53 (0.99 - 2.37) | 0.054 |  |  |  |
| Child-Pugh class | 2.14 (1.22-3.75) | **0.008** |  | 1.17 (0.60 - 2.30) | 0.639 |
| BCLC stage | 1.85 (1.11-3.09) | **0.018** |  | 1.67 (0.93 - 3.00) | 0.086 |
| Tumor number | 1.20 (0.71-2.03) | 0.491 |  |  |  |
| Tumor size of Largest nodule | 1.71 (1.11-2.63) | **0.015** |  | 1.49 (0.91 - 2.44) | 0.115 |
| RBC (10^9^/L) | 0.78 (0.55-1.12) | 0.184 |  |  |  |
| WBC (10^9^/L) | 0.86 (0.75-0.98) | **0.029** |  | 0.93 (0.78 - 1.10) | 0.399 |
| PLT (10^9^/L) | 1.00 (0.99-1.00) | **0.027** |  | 1.00 (0.99 - 1.01) | 0.937 |
| HGB (g/L) | 0.99 (0.98-1.01) | 0.314 |  |  |  |
| AST (U/L) | 1.01 (1.00-1.01) | **0.001** |  | 1.01 (1.00 - 1.01) | 0.104 |
| ALT (U/L) | 1.01 (1.00-1.01) | 0.089 |  |  |  |
| ALP (U/L) | 1.01 (1.00-1.01) | **0.034** |  | 1.00 (0.99 - 1.01) | 0.838 |
| ALB (U/L) | 0.92 (0.88-0.95) | **0.000** |  | 0.95 (0.90 - 1.01) | 0.083 |
| TBIL (μmol/L) | 1.02 (1.01-1.04) | **0.008** |  | 1.01 (0.99 - 1.03) | 0.364 |
| γ-GT (U/L) | 1.00 (1.00-1.01) | 0.055 |  |  |  |
| INR | 6.86 (2.42-19.45) | **0.000** |  | 1.03 (0.20 - 5.46) | 0.970 |
| aMAP score | 2.52 (1.61-3.95) | **0.000** |  | 1.70 (0.92 - 3.13) | 0.088 |
| AFP (ng/mL) | 1.00 (1.00-1.00) | 0.191 |  |  |  |

HR = Hazard Ratio; CI = Confidence Interval. HBV = hepatitis B virus; HCV = hepatitis C virus; BCLC= Barcelona Clinic Liver Cancer; RBC = Red blood cell; WBC = White blood cell; HGB = Hemoglobin; PLT = Platelet; ALT = Alanine aminotransferase; AST = Aspartate aminotransferase; ALB = Albumin; TBIL = Total bilirubin; ALP = Alkaline phosphatase; γ-GT = γ-glutamyl transpeptidase; INR = International Normalized Ratio; AFP = Alpha-fetoprotein.

*p* values in bold are < 0.05.

**Supplementary Table 3.** Comparison of discrimination for tumor characteristics, tumor characteristics +ALBI, and tumor characteristics + categorical aMAP score

|  | Basic model | | |  | | Basic model + ALBI | | |  | | Basic model + categorical aMAP score | | |  |
| --- | --- | --- | --- | --- | --- | --- | --- | --- | --- | --- | --- | --- | --- | --- |
|  | HR (95% CI) | | *p* | |  | | HR (95% CI) | *p* | |  | | HR (95% CI) | *p* | |
| tumor number | 1.81 (0.16-3.74) | | **< 0.001** | |  | | 1.80 (1.32-2.45) | **< 0.001** | |  | | 1.84 (1.35 - 2.50) | **< 0.001** | |
| tumor size of largest nodule | 1.46 (0.14-2.80) | | **0.005** | |  | | 1.45 (1.11-1.90) | **0.006** | |  | | 1.48 (1.14 - 1.93) | **0.004** | |
| aMAP score / ALBI | - | | - | |  | | 1.50 (1.15-1.95) | **0.003** | |  | | 1.57 (1.21 - 2.04) | **0.001** | |
| C-index | 0.582 (0.543-0.620) | Ref. | |  | | 0.603 (0.563-0.644) | | 0.115 | |  | | 0.616 (0.579-0.654) | **0.032** | |

*p* values in bold are < 0.05.
